# Computed Tomography Findings in Pathology‐Confirmed Rhino‐Orbital Mucormycosis During the COVID‐19 Era: A Retrospective Cross‐Sectional Study

**DOI:** 10.1002/hsr2.72997

**Published:** 2026-08-06

**Authors:** Mohsen Naraghi, Hashem Sharifian, Nassib Musa Isiyaku, Amirhossein Babaei

**Affiliations:** ^1^ Department of Otorhinolaryngology‐Head and Neck Surgery Tehran University of Medical Sciences Tehran Iran; ^2^ Otorhinolaryngology Research Center Tehran University of Medical Sciences Tehran Iran; ^3^ Rhinology Research Society Tehran Iran; ^4^ Department of Radiology, Amiralam Hospital Tehran University of Medical Sciences Tehran Iran; ^5^ Department of Radiology, Advanced Diagnostic and Interventional Radiology Research Center (ADIR), Medical Imaging Center, Imam Khomeini Hospital Complex Tehran University of Medical Sciences Tehran Iran

**Keywords:** CT scan, mucormycosis, paranasal sinuses, radiology

## Abstract

**Background and Aims:**

Rhino‐orbital mucormycosis (ROM) emerged as a severe complication during the COVID‐19 era, particularly in immunocompromised patients. Computed tomography (CT) plays a critical role in early detection and assessment of disease extent. This study aimed to describe the CT imaging features of pathology‐confirmed ROM and to evaluate their associations with orbital involvement, particularly proptosis.

**Methods:**

This retrospective, imaging‐based study included patients with histopathologically confirmed ROM who underwent noncontrast paranasal sinus CT. CT scans were systematically reviewed for sinus opacification, bone erosion, laterality, orbital involvement, intracranial extension, and proptosis using predefined operational criteria by a single experienced head and neck radiologist. Proptosis was assessed on axial CT images using the interzygomatic line method. Associations between imaging findings and proptosis were assessed using two‐sided Fisher exact tests with Benjamini–Hochberg false‐discovery‐rate (FDR) correction for multiple comparisons; a Firth penalized logistic regression was fitted as an exploratory multivariable analysis because of data separation. Given the retrospective imaging‐based design, all analyses are regarded as descriptive and hypothesis‐generating.

**Results:**

Of 89 included patients, 47 (52.8%) were male, and 42 (47.2%) were female, with a mean age of 56.25 ± 12.42 years. The most common radiologic finding was opacification of the maxillary sinus (87.6%), followed by the ethmoid sinus (84.3%) and sphenoid sinus (69.7%). In exploratory, unadjusted analyses, ethmoid sinus opacification was more frequent in patients with proptosis than in those without (100.0% vs. 79.4%; two‐sided Fisher exact *p* = 0.034), and patients with sphenoid sinus opacification were older than those without (58.4 ± 10.8 vs. 51.4 ± 14.5 years; Mann–Whitney *p* = 0.028). After Benjamini–Hochberg correction of the proptosis comparisons for multiple testing, no association remained statistically significant (all *q* ≥ 0.25); the age comparison was not part of this correction. All findings are therefore descriptive and hypothesis‐generating.

**Conclusion:**

CT imaging demonstrates characteristic patterns of sinus, bony, and orbital involvement in ROM. The associations observed between specific CT findings and proptosis were exploratory and did not persist after correction for multiple testing; they should therefore be regarded as hypothesis‐generating rather than predictive, while still supporting the value of systematic CT assessment in describing disease extent.

## 1 Introduction

Mucormycosis is a rare but life‐threatening opportunistic fungal infection [1] that primarily affects immunocompromised individuals, with uncontrolled diabetes being the most common predisposing factor [2]. Other risk factors include hematological malignancies, solid organ transplantation, steroid use, and immunodeficiency states [3]. During the COVID‐19 pandemic, an increased incidence of rhino‐orbital mucormycosis has been reported worldwide, attributed to virus‐induced immune dysregulation, corticosteroid therapy, and hyperglycemia. Early recognition of sinonasal involvement is critical to prevent orbital and intracranial complications [4]. In India, the incidence of mucormycosis was stated to have increased 2.1 times during the COVID‐19 pandemic [5]. Similarly, several investigations from Iran have reported a high incidence of mucormycosis in COVID‐19 patients [6, 7].

Imaging, particularly high‐resolution computed tomography (CT), plays a pivotal role in evaluating the extent of disease, including sinus opacification, bony erosions, and orbital involvement [8]. CT is widely accessible, highly sensitive for bone changes, and complements clinical examination in guiding management [9]. Despite the growing number of COVID‐19‐associated cases, systematic data on the CT characteristics of pathology‐confirmed rhino‐orbital mucormycosis remain limited. In the diagnosis of sinonasal mucormycosis, a CT scan is 100% sensitive and 78% specific. Although radiologic findings may mimic malignancy, invasive mycotic infections should be evaluated in the appropriate clinical situations [10].

Despite the growing recognition of COVID‐19‐associated rhino‐orbital mucormycosis, there remains limited systematic data describing the detailed CT imaging characteristics of pathology‐confirmed cases in this context. Most existing reports are either case series or focus on clinical presentation without a comprehensive radiologic assessment. This knowledge gap hinders early diagnosis and appropriate management of patients at risk for orbital and intracranial complications. Therefore, this study aims to systematically evaluate the CT scan findings in patients with rhino‐orbital mucormycosis during the COVID‐19 era.

## 2 Material and Methods

### 2.1 Study Design and Setting

This cross‐sectional study was conducted at Amir Alam Hospital, Tehran, Iran, a tertiary referral center, from October 2020 to October 2021. The study focused on patients referred for evaluation of fungal rhinosinusitis during the COVID‐19 pandemic. The study protocol was approved by the Institutional Review Board (IRB) and Ethics Committee of Tehran University of Medical Sciences (ethic code: IR.TUMS.AMIRALAM.REC.1400.052). The requirement for informed consent was waived by the IRB. All images included in the manuscript are fully de‐identified, and consent for publication of images was waived as per IRB guidelines. The study was conducted in accordance with the ethical principles of the Declaration of Helsinki.

### 2.2 Inclusion and Exclusion Criteria

Patients were included if they met the following criteria: age ≥ 18 years, symptoms of sinusitis, and histopathologically confirmed rhino‐orbital mucormycosis from surgical specimens. Exclusion criteria were: mucormycosis without rhino‐orbital involvement, previous sinus surgery, or incomplete imaging studies.

### 2.3 Study Variables

The primary variables of interest were CT imaging findings, including sinus opacification, bony erosions (floor of the anterior cranial fossa, cribriform plate, lamina papyracea, and pterygopalatine fossa), and orbital involvement (proptosis) (Table 1). Secondary exploratory analyses examined associations between imaging findings and patient age, sex, and proptosis.

**Table 1:** Data dictionary of variables used in the study.

Variable name	Description/definition
Age	Age of patient at time of CT imaging
Sex	Biological sex of patient
Proptosis	Anterior displacement of the globe beyond the interzygomatic line on axial CT
Erosion of the floor of the anterior cranial fossa	CT evidence of erosion or dehiscence of the bony floor of the anterior cranial fossa
Erosion of the cribriform plate	CT evidence of cribriform plate erosion
Erosion of the lamina papyracea	CT evidence of lamina papyracea erosion
Erosion of the pterygopalatine fossa	CT evidence of erosion of the bony margins of the pterygopalatine fossa (the maxillary, palatine, and sphenoid boundaries), with or without obliteration of the fat within the fossa
Opacification of the ethmoid sinus	Opacification (soft‐tissue or fluid attenuation) of the ethmoid sinus on CT
Opacification of the maxillary sinus	Opacification (soft‐tissue or fluid attenuation) of the maxillary sinus on CT
Opacification of the frontal sinus	Opacification (soft‐tissue or fluid attenuation) of the frontal sinus on CT
Opacification of the sphenoid sinus	Opacification (soft‐tissue or fluid attenuation) of the sphenoid sinus on CT

### 2.4 Data Collection

CT scans were reviewed and interpreted by a single experienced head and neck radiologist, who applied the predefined operational criteria detailed below and was blinded to the histopathological diagnosis and to the clinical proptosis status during image review. Because all images were assessed by one reader, interobserver reliability could not be evaluated; this is acknowledged as a limitation. Patient demographic data, including age and sex, were extracted from hospital records. Data collection followed a standardized protocol to ensure consistency and reliability.

### 2.5 CT Imaging and Interpretation Protocol

All patients underwent noncontrast CT scanning using a Siemens SOMATOM Emotion 16‐slice scanner (Siemens, Erlangen, Germany). Scan parameters included spiral acquisition with 0.6 mm slice thickness, 110 kVp, 70 mAs, pitch factor of 1.5, and 0.75 mm acquisition design. Axial and coronal images were reconstructed using bone and soft tissue kernels with a matrix of 512 × 512 and a field of view (FOV) of 200–250 mm. Window and level presets were adjusted for optimal bone and soft tissue assessment. Multiplanar reconstructions were routinely generated to evaluate sinus anatomy, bony erosion, and orbital or intracranial extension.

**Figure 1:**
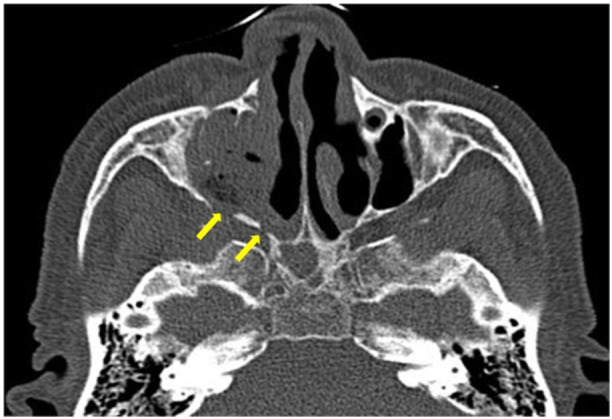
Axial noncontrast computed tomography (CT) image in bone window demonstrating involvement of the bony margins of the right pterygopalatine fossa. Arrows indicate areas of cortical erosion.

**Figure 2:**
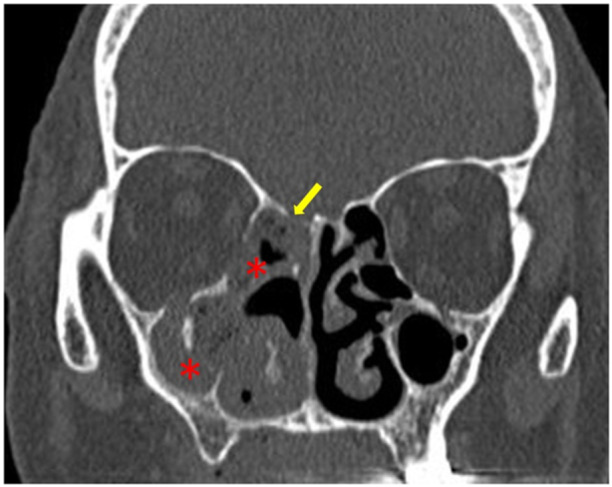
Coronal noncontrast CT image displayed in soft tissue and bone windows showing opacification of the right‐sided paranasal sinuses (*) with erosion of the cribriform plate (arrow).

**Figure 3:**
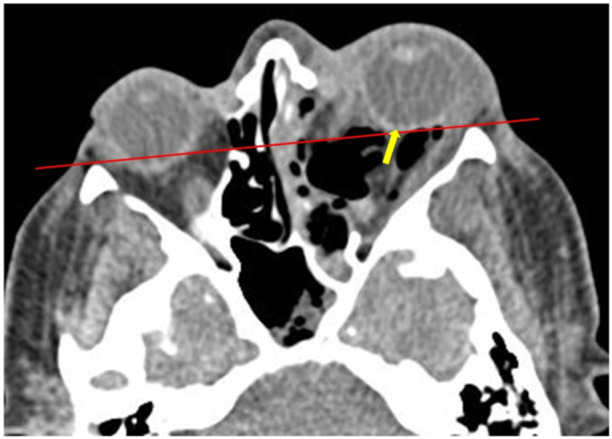
Axial noncontrast CT image in soft tissue window demonstrating left orbital involvement with proptosis. The arrow indicates anterior displacement of the globe beyond the interzygomatic line (red line).

All imaging variables were defined using standardized operational criteria to ensure reproducibility and consistency. Sinus opacification was classified as partial or complete replacement of normally aerated sinus by soft tissue density on CT. Bone erosion was identified as cortical thinning, loss, or discontinuity of bony walls, including the floor of the anterior cranial fossa, cribriform plate, lamina papyracea, and the bony margins of the pterygopalatine fossa (Figures 1, 2). Proptosis was assessed on axial CT images using the interzygomatic line method. On the axial section passing through the lens, a baseline (the interzygomatic line) was drawn connecting the anterior margins of both zygomatic processes, and the perpendicular distance from this line to the anterior surface of the globe was measured; a distance of ≥ 21 mm was considered indicative of proptosis (Figure 3). All variables were recorded as present or absent according to these criteria.

### 2.6 Diagnostic Confirmation

All cases were confirmed by histopathological examination of surgical sinus specimens. Tissue samples were fixed in 10% formalin, embedded in paraffin, and sectioned for hematoxylin and eosin staining. Grocott's methenamine silver (GMS) and periodic acid‐Schiff (PAS) staining were performed to highlight fungal hyphae. Mucormycosis was diagnosed based on the presence of broad, nonseptate hyphae with right‐angle branching invading tissue. Pathologic review was conducted by an experienced pathologist, ensuring accurate identification of fungal elements and confirming rhino‐orbital involvement.

### 2.7 Statistical Analysis

Data analysis was performed using IBM SPSS Statistics version 25 (IBM Corporation, Armonk, NY, USA), with the Firth penalized logistic regression fitted in R version 4.3 (logistf package). Continuous variables were reported as mean ± standard deviation (SD), and categorical variables as counts and percentages. Associations between categorical variables and proptosis were evaluated using two‐sided Fisher exact tests, and unadjusted odds ratios (ORs) with 95% confidence intervals (CIs) were calculated from the corresponding 2 × 2 tables. Differences in age across imaging findings were assessed with the Mann–Whitney *U*‐test. To address multiplicity, the family of associations with proptosis was corrected using the Benjamini–Hochberg false‐discovery‐rate (FDR) procedure, and FDR‐adjusted *q* values are reported. As an exploratory multivariable analysis, variables with *p* < 0.2 in the unadjusted comparison with proptosis (erosion of the lamina papyracea, erosion of the pterygopalatine fossa, and ethmoid sinus opacification) were entered into a Firth penalized logistic regression. The Firth method was chosen because standard maximum‐likelihood logistic regression did not converge for ethmoid sinus opacification owing to complete separation (all patients with proptosis had ethmoid opacification); Firth penalization yields finite estimates and profile‐penalized‐likelihood CIs under these conditions. Adjusted ORs with 95% profile‐likelihood CIs and penalized likelihood‐ratio *P*‐values are reported. Given the retrospective imaging‐based design, the limited number of events, and the exploratory nature of the regression, all inferential results are interpreted as descriptive and hypothesis‐generating rather than as established predictors. Statistical significance was set at *p* < 0.05.

## 3 Results

A total of 89 patients were included in the study. During the study period, consecutive patients referred to the center for evaluation of fungal rhinosinusitis were screened against the eligibility criteria. Patients were retained only if they were aged ≥ 18 years, had symptoms of sinusitis, and had rhino‐orbital mucormycosis confirmed on histopathology of surgical specimens; those with mucormycosis lacking rhino‐orbital involvement, a history of previous sinus surgery, or incomplete imaging studies were excluded. Application of these criteria yielded the final analytic cohort of 89 patients, all of whom had both a histopathologic diagnosis and an evaluable noncontrast paranasal sinus CT, and all 89 were included in the analyses reported below. Among them, 47 (52.8%) were male, and 42 (47.2%) were female, with a mean age of 56.25 ± 12.42 years.

**Figure 4:**
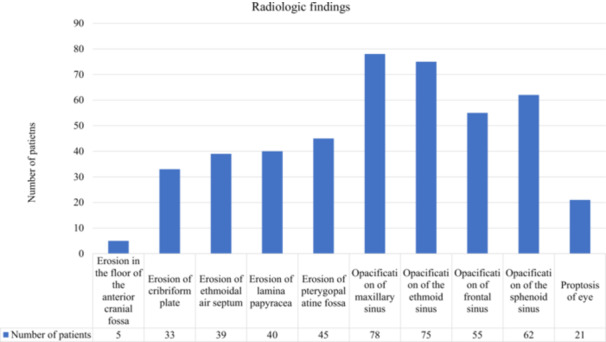
Summary of radiologic findings in the study.

The most common radiologic finding was opacification of the maxillary sinus (87.6%), followed by opacification of the ethmoid sinus (84.3%) and sphenoid sinus (69.7%). The least common finding was erosion of the floor of the anterior cranial fossa (5.6%) (Figure 4).

Analysis of the relationship between patient age and CT findings revealed that patients with opacification of the sphenoid sinus were older (58.39 ± 10.83 years) compared to those without sphenoid sinus opacification (51.37 ± 14.54 years; Mann–Whitney *p* = 0.028); this exploratory age comparison was not adjusted for multiple testing (Table 2). No significant age differences were observed for other sinus opacifications or bony erosions.

**Table 2:** Relation of radiologic findings with patients' age.

Variable		Age; mean (SD)	*p*‐value*
Sex	Male	55.89 (11.82)	0.684
	Female	56.67 (13.20)	
Erosion in the floor of the anterior cranial fossa	Yes	61.60 (5.59)	0.265
	No	55.94 (12.66)	
Erosion of cribriform plate	Yes	56.70 (10.43)	0.885
	No	56.00 (13.54)	
Erosion of lamina papyracea	Yes	57.30 (10.28)	0.336
	No	55.41 (13.98)	
Erosion of pterygopalatine fossa	Yes	57.64 (10.55)	0.289
	No	54.84 (14.07)	
Opacification of maxillary sinus	Yes	55.81 (12.74)	0.477
	No	59.45 (9.77)	
Opacification of the ethmoid sinus	Yes	56.93 (11.08)	0.327
	No	52.64 (18.15)	
Opacification of frontal sinus	Yes	57.24 (11.98)	0.270
	No	54.68 (13.13)	
Opacification of the sphenoid sinus	Yes	58.39 (10.83)	0.028
	No	51.37 (14.54)	
Proptosis of eye	Yes	54.62 (10.73)	0.523
	No	56.76 (12.93)	

* Mann–Whitney *U*‐test

No statistically significant differences in CT findings were observed between male and female patients (Table 2).

Proptosis was present in 21 (23.6%) patients. In unadjusted comparisons, ethmoid sinus opacification was more frequent among patients with proptosis than among those without (100.0% vs. 79.4%; two‐sided Fisher exact *p* = 0.034; the unadjusted OR was not estimable because all patients with proptosis had ethmoid opacification, producing complete separation). Erosion of the lamina papyracea (61.9% vs. 39.7%; OR 2.47, 95% CI: 0.90–6.75; *p* = 0.085) and erosion of the pterygopalatine fossa (33.3% vs. 55.9%; OR 0.39, 95% CI: 0.14–1.10; *p* = 0.085) showed associations of borderline magnitude, whereas the remaining sinus opacifications and bony erosions were not associated with proptosis (Table 3). After Benjamini–Hochberg correction for the nine comparisons, none of these associations remained statistically significant (all *q* ≥ 0.25).

**Table 3:** Association of patient sex and radiologic findings with proptosis.

Variable	Proptosis present (*N* = 21); *n* (%)	Proptosis absent (*N* = 68); *n* (%)	*p*‐value*	Unadjusted OR (95% CI)^a^	FDR *q* b
Sex (male); *n* (%)	9 (42.9)	38 (55.9)	0.327	0.59 (0.22–1.59)	0.735
Erosion of the floor of the anterior cranial fossa; *n* (%)	2 (9.5)	3 (4.4)	0.588	2.28 (0.35–14.66)	0.886
Erosion of the cribriform plate; *n* (%)	8 (38.1)	25 (36.8)	1.000	1.06 (0.39–2.90)	1.000
Erosion of the lamina papyracea; *n* (%)	13 (61.9)	27 (39.7)	0.085	2.47 (0.90–6.75)	0.254
Erosion of the pterygopalatine fossa; *n* (%)	7 (33.3)	38 (55.9)	0.085	0.39 (0.14–1.10)	0.254
Opacification of the maxillary sinus; *n* (%)	18 (85.7)	60 (88.2)	0.717	0.80 (0.19–3.34)	0.897
Opacification of the ethmoid sinus; *n* (%)	21 (100.0)	54 (79.4)	0.034	NE§	0.254
Opacification of the frontal sinus; *n* (%)	14 (66.7)	41 (60.3)	0.798	1.32 (0.47–3.69)	0.897
Opacification of the sphenoid sinus; *n* (%)	16 (76.2)	46 (67.6)	0.590	1.53 (0.50–4.72)	0.886

* *p*‐values from two‐sided Fisher exact tests.

^a^ OR = unadjusted odds ratio estimated from the 2 × 2 table; CI, 95% confidence interval.

^b^ *q* = Benjamini–Hochberg false‐discovery‐rate‐adjusted *p*‐value across the nine comparisons. § NE, not estimable owing to complete separation (all patients with proptosis had ethmoid sinus opacification).

Because all patients with proptosis had ethmoid sinus opacification, the data showed complete separation, and a standard maximum‐likelihood logistic model did not converge for this variable (the unpenalized model produced an implausibly large coefficient with an undefined standard error). An exploratory Firth penalized logistic regression (Table 4) was therefore fitted, including erosion of the lamina papyracea, erosion of the pterygopalatine fossa, and ethmoid sinus opacification (the variables with *p* < 0.2 in the unadjusted analysis). None of the adjusted associations reached statistical significance: erosion of the lamina papyracea (adjusted OR = 2.15, 95% CI: 0.77–6.36, *p* = 0.145), erosion of the pterygopalatine fossa (adjusted OR = 0.40, 95% CI: 0.13–1.11, *p* = 0.078), and ethmoid sinus opacification (adjusted OR = 7.58, 95% CI: 0.87–998, *p* = 0.071). The extremely wide confidence interval for ethmoid opacification reflects the underlying separation and indicates that its adjusted effect cannot be estimated with precision in this sample (apparent *c*‐statistic = 0.72; 21 events for three covariates). These exploratory results are compatible with an association between ethmoid and lamina involvement and proptosis but do not establish one and should be regarded as hypothesis‐generating.

**Table 4:** Exploratory multivariable Firth penalized logistic regression for proptosis.

Variable	*B*	SE	Adjusted OR (95% CI)	*p*‐value*
Erosion of the lamina papyracea	0.764	0.537	2.15 (0.77–6.36)	0.145
Erosion of the pterygopalatine fossa	−0.928	0.540	0.40 (0.13–1.11)	0.078
Opacification of the ethmoid sinus	2.025	1.510	7.58 (0.87–997.8)	0.071
Intercept	−2.936	—	—	0.002

* *p*‐values from penalized likelihood‐ratio tests. B, penalized regression coefficient; SE, standard error; OR, adjusted odds ratio with 95% profile‐likelihood confidence interval. A Firth penalized logistic regression was used because standard maximum‐likelihood estimation did not converge owing to complete separation (all patients with proptosis had ethmoid sinus opacification). Candidate covariates were those with *p* < 0.2 in the unadjusted comparison with proptosis. The model included 21 events for three covariates, and the apparent c‐statistic (area under the ROC curve) was 0.72. These results are exploratory and hypothesis‐generating, and no covariate reached statistical significance.

## 4 Discussion

Rhino‐orbital mucormycosis is a rare but life‐threatening fungal infection, and systematic data on its imaging characteristics during the COVID‐19 era remain limited. Most previous studies have focused on small case series or included patients without histopathologic confirmation, leaving a gap in understanding the typical CT features in confirmed cases. The present study aimed to characterize the CT findings in patients with pathology‐confirmed rhino‐orbital mucormycosis, with a focus on sinus opacification, bony erosion, and orbital involvement, and to explore associations with patient age, sex, and proptosis. By providing a detailed, cross‐sectional analysis of 89 confirmed cases, this study contextualizes imaging patterns that may facilitate early recognition and timely management of this aggressive infection.

The results of our study revealed that the most common radiologic finding was opacification of the maxillary sinus, followed by opacification of the ethmoid sinus. In exploratory analyses, patients with sphenoid sinus opacification were older, and ethmoid sinus opacification was more frequent among patients with proptosis; however, neither association remained statistically significant after correction for multiple comparisons, so both are best regarded as descriptive observations rather than confirmed effects.

Several conditions may enhance the possibility of rhino‐orbital mucormycosis following COVID‐19: endothelial dysfunction due to the virus, high blood sugar, hyperferritinemia and iron acquisition from the host, and immune dysfunction owing to corticosteroid use increase the risk of rhino‐orbital mucormycosis infection. Another risk factor that might be responsible for the current increase of mucormycosis in COVID‐19 individuals can be oxygen humidifiers. They were widely used in COVID‐19 patients with hypoxia. They can be a factor in spreading possible nosocomial microbes by generating aerosol particles, which go into the lungs immediately after inhalation [11, 12, 13].

Clinical presentation of rhino‐orbital mucormycosis may start with nasal turbinate necrosis, palatal necrosis, or sinus necrosis and could quickly progress to the orbit before involving intracranial organs. The common complaints of patients are fever, headache, amaurosis, epistaxis, proptosis, facial paralysis, and signs of trigeminal nerve invasion. Cavernous sinus thrombosis and cranial invasion may be consequences of untreated rhino‐orbital mucormycosis [14].

In our study, the mean age of patients was 56.25 ± 12.42 years. It was in line with previously published manuscripts [15, 16].

CT scan is a cost‐effective technique for the detection of bone erosions. Magnetic resonance imaging (MRI) with intravenous contrast injection is beneficial in showing intracranial spread, perineural extension, and subtle fat stranding in the facial, orbital, and retroantral areas [17, 18]. A CT scan detects mucosal thickening and hyperdense secretions in the involved sinuses in the initial phases. Subsequently, medial orbital wall destruction and invasion of the rectus muscles, orbital apex, and ipsilateral cavernous sinus may be detected [19].

In our study, the most common radiologic finding was opacification of the maxillary sinus (87.6%), followed by opacification of the ethmoid sinus (84.3%) and opacification of the sphenoid sinus (69.7%). The least common radiologic presentation was erosion in the floor of the anterior cranial fossa (5.6%). In a study of the radiographic manifestation of 43 individuals with mucormycosis, Therakathu et al. reported that 79% of patients had unilateral sinus involvement and only 2% presented with pan sinusitis. They also reported that the orbital wall was the most common part of bony erosions [20]. In contrast, we found that erosion of the pterygopalatine fossa was the most commonly involved bony structure, which could be explained by the delayed diagnosis of the disease. Singhal et al. stated the combination of maxillary, ethmoid, and sphenoid sinus involvement in 18 patients (72%). All individuals had periantral involvement, and 14 had concomitant orbital involvement. Accompanying sinonasal, periantral, and orbital involvement with tissue necrosis was typical of acute invasive mucormycosis [21]. The aggressive nature of the infection through the involvement of periantral and orbital fat was described previously [22].

In our study, erosion of the lamina papyracea did not differ in patients with and without proptosis. It can be explained by the fact that orbital extension also happens via the nasolacrimal duct, anterior and posterior ethmoid orifices, or dehiscent lamina papyracea [21, 23].

The predominance of maxillary and ethmoid sinus opacification observed in this study aligns with prior reports, reflecting the typical initial sites of fungal invasion in rhino‐orbital mucormycosis. The association of sphenoid sinus involvement with older age may suggest a delayed or more extensive disease course in this population, which has been less consistently reported in the literature. The higher frequency of ethmoid sinus opacification among patients with proptosis is anatomically plausible, given the proximity of the ethmoid sinus to the orbit, and is consistent with earlier reports linking ethmoid involvement to orbital extension. In our data, however, this association did not survive correction for multiple testing and could not be quantified precisely because all patients with proptosis had ethmoid opacification (complete separation); it should therefore be interpreted as a descriptive, hypothesis‐generating observation rather than as an established predictor of orbital involvement. Compared with previous studies, the frequency of bony erosions in our cohort was somewhat lower, potentially due to early imaging or differences in disease severity.

Our findings should also be positioned relative to the predominantly MRI‐based literature on orbital and extrasinus extension. Most prior studies characterizing orbital, cavernous sinus, perineural, and intracranial involvement in rhino‐orbital mucormycosis have relied on MRI or on mixed CT and MRI protocols, which differ from the CT‐only approach used here. MRI offers superior soft‐tissue contrast and is generally more sensitive than CT for detecting early orbital infiltration, perineural spread, and intracranial extension, whereas CT is more widely available in acute settings and better demonstrates bony erosion and sinus opacification. Consequently, series based on MRI or combined modalities may report higher rates of orbital and intracranial involvement than the present CT‐based cohort, and direct comparison across studies is further complicated by differing modalities and by varying definitions of orbital involvement. Our results should therefore be understood as reflecting CT‐detectable disease, and the absence of an MRI correlate is an acknowledged limitation when interpreting these findings within the wider evidence base.

The strengths of our study include its acceptable sample size, systematic evaluation of pathology‐confirmed cases, and detailed CT assessment of sinus and orbital involvement. Limitations include its retrospective, imaging‐based design, which resulted in incomplete clinical and demographic data, such as chief complaints, COVID‐19 severity, clinical presentation, and disease duration. The study was conducted at a single center with a pathology‐confirmed cohort, which may introduce selection bias and skew toward more advanced disease. Treatment approaches were not analyzed, as the focus was on CT findings. All images were reviewed by a single radiologist, which may introduce observer bias. Additionally, the absence of a control group and lack of longitudinal follow‐up, including clinical course, vision outcomes, and surgical interventions, limit the generalizability and scope of the findings. Furthermore, because CT was the sole imaging modality, the extent of orbital, perineural, and intracranial soft‐tissue involvement may have been underestimated relative to MRI, which has greater soft‐tissue contrast. As all images were assessed by a single reader, no formal interobserver reliability could be calculated, and observer agreement therefore could not be quantified. Finally, the comparative analyses were exploratory in nature: the relatively small number of patients with proptosis, the multiple unadjusted univariable comparisons, and the complete separation affecting the multivariable model increase the risk of spurious or unstable associations. For these reasons, the reported associations should be regarded as descriptive and hypothesis‐generating rather than confirmatory or predictive.

## 5 Conclusion

In this retrospective, cross‐sectional study of patients with pathology‐confirmed rhino‐orbital mucormycosis, sinus opacification, particularly of the maxillary sinus, was the most common CT finding, while bony erosions were less frequent. In exploratory, unadjusted analyses, sphenoid sinus opacification was associated with older age, and ethmoid sinus opacification was more frequent among patients with proptosis; however, no association remained statistically significant after correction for multiple testing, and the multivariable model was limited by complete separation. These observations should therefore be interpreted as descriptive and hypothesis‐generating rather than as established or independent predictors of orbital involvement. They nonetheless reinforce the value of careful CT evaluation of the sinuses and orbit in the diagnostic assessment of rhino‐orbital mucormycosis during the COVID‐19 era, and they may help inform the design of larger, prospective, multimodality studies.

## Author Contributions

**Mohsen Naraghi:** methodology, conceptualization, investigation, funding acquisition, supervision, writing – review and editing. **Hashem Sharifian:** methodology, writing – review and editing, data curation. **Nassib Musa Isiyaku:** data curation, writing – review and editing, investigation. **Amirhossein Babaei:** writing – original draft, data curation, formal analysis.

## Funding

The authors have nothing to report.

## Conflicts of Interest

The authors declare no conflicts of interest.

## Author Declaration

All authors have read and approved the final version of the manuscript. The corresponding author, Mohsen Naraghi, had full access to all of the data in this study and takes complete responsibility for the integrity of the data and the accuracy of the data analysis.

## Transparency Statement

The lead author and manuscript guarantor, Mohsen Naraghi, affirms that this manuscript is an honest, accurate, and transparent account of the study being reported; that no important aspects of the study have been omitted; and that any discrepancies from the study as planned (and, if relevant, registered) have been explained.

## Data Availability

The data that support the findings of this study are available from the corresponding author upon reasonable request.
